# Common sequence variants in the *LOXL1* gene in pigment dispersion syndrome and pigmentary glaucoma

**DOI:** 10.1186/1471-2415-14-52

**Published:** 2014-04-16

**Authors:** Emiliano Giardina, Francesco Oddone, Tiziana Lepre, Marco Centofanti, Cristina Peconi, Lucia Tanga, Luciano Quaranta, Paolo Frezzotti, Giuseppe Novelli, Gianluca Manni

**Affiliations:** 1 Department of Biomedicine and Prevention, School of Medicine, University of Rome “Tor Vergata” and Fondazione PTV “Policlinico Tor Vergata”, Rome, Italy; 2 Laboratory of Molecular Genetics UILDM, Fondazione Santa Lucia, Roma, Italy; 3 Fondazione G.B. Bietti-IRCCS, Via Livenza 3, 00198 Rome, Italy; 4 DSCMT, University of Tor Vergata, Rome, Italy; 5 Glaucoma Unit, University of Brescia, Brescia, Italy; 6 Department of Surgery, University of Siena, Siena SI, Italy

**Keywords:** Pigment dispersion syndrome, Pigmentary glaucoma, Genetics, *LOXL1*

## Abstract

**Background:**

Single nucleotide polymorphisms (SNPs) within the *LOXL1* gene are associated with pseudoesfoliation syndrome and pseudoesfoliation glaucoma. The aim of our study is to investigate a potential involvement of *LOXL1* gene in the pathogenesis of pigment dispersion syndrome (PDS) and pigmentary glaucoma (PG).

**Methods:**

A cohort of Caucasian origin of 84 unrelated and clinically well-characterised patients with PDS/PG and 200 control subjects were included in the study. Genomic DNA from whole blood was extracted and the coding and regulatory regions of *LOXL1* gene were risequenced in both patients and controls to identify unknown sequence variations. Genotype and haplotype analysis were performed with UNPHASED software. The expression levels of *LOXL1* were determined on c-DNA from peripheral blood lymphocytes by quantitative real-time RT-PCR.

**Results:**

A significant allele association was detected for SNP rs2304722 within the fifth intron of *LOXL1* (Odds ratio (OR = 2.43, p-value = 3,05e-2). Haplotype analysis revealed the existence of risk and protective haplotypes associated with PG-PDS (OR = 3.35; p-value = 1.00e-5 and OR = 3.35; p-value = 1.00e-4, respectively). Expression analysis suggests that associated haplotypes can regulate the expression level *LOXL1*.

**Conclusions:**

Haplotypes of *LOXL1* are associated with PG-PDS independently from rs1048661, leading to a differential expression of the transcript.

## Background

Glaucoma, the leading global cause of irreversible visual impairment and blindness, is a group of chronic, progressive optic neuropathies that have in common characteristic morphological changes at the optic nerve head and retinal nerve fibre layer [1]. The cause of glaucomatous neuropathy is unknown; previous studies have suggested that multiple factors, including multiple genetic factors, might be responsible for this condition. Pigment dispersion syndrome (PDS) and pigmentary glaucoma (PG) are the most common non-traumatic causes of glaucoma in young adults and are characterised by disruption of the iris pigment epithelium and deposition of abnormal pigment granules throughout the anterior segment, which results in aqueous outflow obstruction [2].

Like the majority of human diseases PG and PDS show a familial aggregation that does not usually follow Mendelian family patterns but is caused by an unknown number of multiple genes, usually interacting with various environmental factors. A single susceptibility locus to PDS has been mapped on chromosome 7q35-q36 but the candidate gene is yet to be identified [3].

Pseudoexfoliation syndrome (PEXS) is the most common identifiable cause of open-angle glaucoma, accounting for approximately 25% of all open-angle glaucoma worldwide [4]. It is an age-related systemic disease of the extracellular matrix characterized by the multifocal production and progressive accumulation of a fibrillar extracellular material in intra and extra-ocular tissues that is either the result of an excessive production or insufficient breakdown or both [5].

Pseudoexfoliation syndrome (PEXS) is the most common identifiable cause of openangle glaucoma, accounting for approximately 25% of all open-angle glaucoma worldwide [4]. It is an age-related systemic disease of the extracellular matrix characterised by the multifocal production and progressive accumulation of a fibrillar extracellular material in intra- and extraocular tissues, which is either the result of excessive production, insufficient breakdown or both [5]. PEXS and PDS are two disorders that can produce secondary glaucoma through trabecular blockage [2,6] Secondary open-angle glaucoma because of PEXS (PEXG) develops from the deposition of exfoliation material in the trabecular meshwork, leading to elevated intraocular pressure (IOP) and consequently glaucomatous optic neuropathy [7].

Recently, a genome-wide association study demonstrated that one intronic single nucleotide polymorphism (SNP) (rs2165241) and two non-synonymous coding SNPs (rs1048661 (R141L) and rs3825942 (G153D)), located in the first exon of the *LOXL1* gene (15q24-q25), confer risk to PEXG through PEXS in the Icelandic and Swedish populations [8]. Several following studies replicated the association of these two nonsynonymous SNPs with exfoliation syndrome (XFS) in different populations [7,9-14].

The aim of our study was the mutational analysis of the coding and regulatory regions of *LOXL1* to disclose unknown variants and/or haplotypes associated with the development of pigment PDS and PG.

## Methods

### Study subjects

Unrelated Italian patients with clinically well-characterised PDS/PG and healthy control subjects were recruited at the moment of referral at the glaucoma unit of the Bietti Eye Foundation IRCCS, Rome, Italy and the ophthalmology clinic of the University of Tor Vergata, Rome, Italy. The study was approved by the local ethics committee (Ethics Committee of the University of Tor Vergata, R. S. 91.10, 24/09/2010) and adhered to the tenets of the Declaration of Helsinki and local law.

Written informed consent was obtained before enrolment from all subjects. After the collection of demographic and historical data, all enrolled subjects underwent a complete detailed ophthalmological examination including (i) corrected visual acuity (ETDRS), (ii) slit lamp biomicroscopy examination of the anterior segment, (iii) gonioscopic evaluation of the anterior chamber angle using a one mirror high magnification gonio lens (MagnaView Gonio laser lens), (iv) measurement of IOP using Goldmann applanation tonometry and (v) indirect ophthalmoscopy using a 90D Volk’s lens. Moreover, the visual field (VF) was tested in all subjects using the Humphrey Field Analyzer 24-2 test pattern and SITA standard threshold strategy.

Subjects with PDS were defined as those with an open angle at gonioscopy, trabecular meshwork pigmentation, the presence of a Krukenberg spindle and midperipheral iris transillumination. Subjects with PG were defined as those with a documented history of IOP > 24 mmHg, optic disc and confirmed VF damage (defined as a glaucoma hemifield test result outside normal limits, MD and PSD outside 95% confidence limits and a cluster of at least three points with *P* < 0.05 in the pattern deviation plot, with one of each with *P* < 0.01 affecting the same hemifield).

Common exclusion criteria for PDS/PG were a history of ocular surgery or traumas, any active ocular pathology different from glaucoma, PEXS or PEXG, other causes of secondary glaucoma or a history of long-term use of topical or systemic steroids.

Healthy subjects with no history of IOP > 22 mmHg in both eyes, open angle at gonioscopy, normal anterior segment, normal appearance of optic disc and normal VF were recruited as controls.

### Genotyping

Genomic DNA was extracted from peripheral white blood cells of all subjects using the QIAamp DNA blood mini kit (Qiagen Ltd, Valencia, CA, USA). The coding and regulatory regions of *LOXL1* were resequenced in both patients (n = 84) and healthy controls (n = 100) using sequence specific primers (Table 1). PCR products were purified using an ultraclean PCR purification kit (DyeEx Kits, Qiagen, Hilden, Germany) and sequenced in an ABI 3130 × l sequencer using a Big Dye 3.1 terminator ready reaction kit (Applied Biosystems, California, USA). DNA sequences were analysed using the sequencing analysis 3.0 software package (Applied Biosystems, California, USA). The genotyping of rs1048661, rs3825942 and rs2304722 was performed in an extended control cohort (n = 100) by TaqMan assays. Reactions were run in an AB7500 (Applied Biosystems, California, USA) and interpreted using Sequence Detection System (SDS) 2.1 software.

**Table 1:** **Self-designed primers used for *LOXL1 *** **resequencing**

	**Forward 5′-3′**	**Reverse 5′-3′**	**Amplicons size (bp)**
Exon 1	GCAGGTGTACAGCTTGCTCA	ACACGAAACCCTGGTCGTG	420
Intron 1	GCAGAAGGGCGATTATAGC	CATGTGAGTACACAGCTTG	392
Exon 2	CTGATGCTCTCAATGCATGC	CTTGCAGACAGCCTAGCTG	228
Exon 3	CATGCTGGGTTCTGGTGTCA	CTAATCCAGTATCTGTCTC	258
Exon 4	GACTAGGCCCTCTTCTTTCTC	CGCTCTTTGCCTCCCCAAC	224
Exon 5	CAGAAACTCCTGAAGGTGGG	GCTGAAGCTTCTTTCAGGAAC	240
Exon 6	CTCTCTGTCTGTCTGTCTGC	CTGCGTTCGGCCATCAAGG	304
Exon 7	GAAATGTAGGCCCATGCTG	GAAGGATGATGCCTAAGGAC	486

### Real-time PCR

The generation of first-strand cDNA was performed using a high capacity DNA reverse transcription kit (Applied Biosystems) according to the manufacturer’s instructions, and the reverse transcriptase reaction products were used for quantitative real-time PCR, which was run in an AB7500 (Applied Biosystems) using TaqMan predesigned probes (Hs00935937_m1).

The amount of mRNA for *LOXL1* in each sample was normalised to the amount of mRNA of the beta 2-tubulin reference gene in the same sample. Relative mRNA expression levels of all examined genes were measured using the comparative 2 ΔΔCT Ct method. Each plate contained three positive controls (samples previously confirmed by direct sequencing as both heterozygous and homozygous) and two negative controls.

### Statistical analysis

Statistical analyses were performed using a standard 2 × 2 table and Fisher’s exact tests. Allele and genotype frequencies of each sequence variant were compared between patients and healthy controls. Haplotype analysis in unrelated samples was performed using the software UNPHASED, available at https://sites.google.com/site/fdudbridge/software/unphased-3-1. Odds ratios (ORs) were calculated using the online software “calculator for confidence intervals of OR in an unmatched case control study” available at http://www.hutchon.net/ConfidOR.htm. Linkage disequilibrium (LD) was evaluated by LD plotter available at http://www.pharmgat.org.

### RNA preparation

Total RNA was extracted from lymphocyte cells with an RNAeasy mini kit (Qiagen, Hilden, Germany) as per the manufacturer’s instructions. RNA was then eluted in 30 μl of RNase-free water and stored at –80°C.

## Results

Altogether, 84 PDS/PG patients (mean age 48.0 ± 4.9 years, 40 females and 44 males), and 200 healthy control subjects (mean age 46.7 ± 5.2 years, 97 males and103 females) were enrolled in this study.

The regulatory and coding regions of *LOXL1* were resequenced in all enrolled PG/PDS control subjects to identify sequence variants. Table 2 reports a list of identified sequence variants and allele and genotype frequency in our cohort of patients and controls. No departure from the Hardy–Weinberg equilibrium was detected for all SNPs identified. Allele frequencies of SNPs rs1048661 and rs3825942, associated to pseudoexfoliation syndrome and pseudoexfoliation glaucoma [8], showed weak differences between cases and controls, although they didnot reach significance (Table 2). We failed to reveal differences in allele and genotype frequency for rs2165241. Notably, a significant association for rs2304722 (intron 5) was detected in PG/PDS patients (Table 2) with the T allele showing a frequency of 0.90 in PG/PDS patients and 0.80 in control subjects (OR = 2.43, pvalue = 3.05e-2).

**Table 2:** Location and allele frequencies of sequence variation identified in PDS/PG cases and controls

**Position**	**SNP**	**Variation**	PDS*/PG^**†**^	**Controls**	***P***
Exon 1	rs1048661	G/T	G = 0.66	G = 0.71	NS^‡^
			T = 0.34	T = 0.29	
Exon 1	rs3825942	G/A	G = 0.84	G = 0.8	NS
			A = 0.16	A = 0.2	
Exon 1	rs2165241	C/T	C = 0.51	C = 0.50	NS
			T = 0.49	T = 0.50	
Intron 5	rs2304722	C/T	C = 0.09	C = 0.21	1.62e-4
			T = 0.91	T = 0.79	
Exon 7	rs8818	G/C	G = 0.69	G = 0.68	NS
			C = 0.31	C = 0.32	
Exon 7	rs3522	C/T	T = 0.44	T = 0.45	NS
			C = 0.56	C = 0.55	

*Pigmentary Dispersion Syndrome; ^†^Pigmentary Glaucoma; ^‡^Not significant.

**Figure 1:**
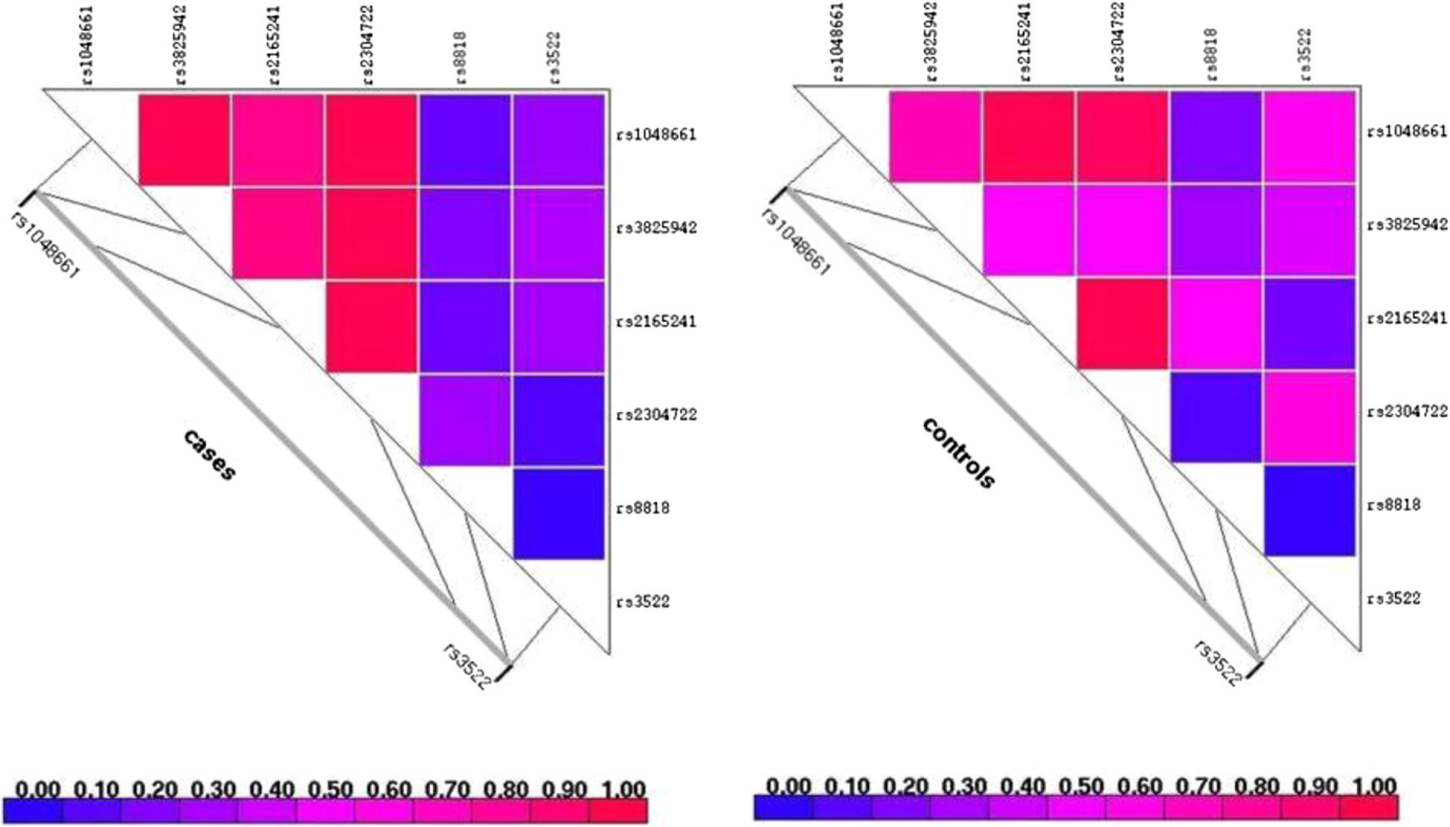
**Linkage Disequilibrium patterns in both cases (left) and controls (right).** The extent of LD is higher in cases chromosomes than in control chromosomes. Cases: rs1048661-rs3825942 D’ = 1; rs3825942- rs2304722 D’ = 1; rs1048661-rs2304722 D’ = 1. Controls: rs1048661-rs3825942 D’ = 0.73; rs3825942- rs2304722 D’ = 0.51; rs1048661-rs2304722 D’ = 0.96.

Pairwise LD analysis for all identified SNPs revealed differences in LD patterns between cases and controls (Figure 1). LD patterns suggested the existence of a conserved block between markers rs1048661 and rs2165241 associated with the disease. The extent of LD was different between the case and control groups, confirming the region between rs1048661 and rs2304722 as a locus of putative association.

Afterwards, a fine mapping of specific haplotype subsets within the block was performed to narrow the minimal associated chromosomal region. As expected, haplotype analysis confirmed the LD findings revealing a highly associated haplotype for markers rs1048661, rs3825942 and rs2304722. Haplotype harbouring of the T allele of rs1048661, G allele of rs3825942 and T allele of rs2304722 was significantly over-represented only in PG/PDS cases (OR = 3.35; p-value = 1.0e-5) (Table 3). Conversely, haplotype harbouring of the T allele of rs1048661, G allele of rs3825942 and C allele of rs2304722 was significantly under-represented in PG/PDS chromosomes (OR = 3.35; p-value = 1.0e-4). The further dissection of haplotype distribution in case and control chromosomes revealed that risk and protective haplotypes shared the same alleles at loci rs1048661 and rs3825942, suggesting a role for rs2304722. By contrast, haplotypes GGT and GAT, harbouring the T allele of rs2304722, were unassociated in our cohort, ruling out a direct functional role for this allele.

**Table 3:** ***LOXL1 *** **Haplotypes in PDS/PG cases and controls**

Haplotype*	**No. (%) of PDS/PG patients**	**No. (%) of control subjects**	**OR (95% CI)**	***P***
GGT	0.50	0.51	0.96 (0.66-1.40)	NS
GAT	0.16	0.20	0.79 (0.48-1.31)	NS
TGC	0.09	0.20	0.41 (0.23-0.76)	1e-4
TGT	0.25	0.09	3.35 (2.02-5.58)	1e-5

*Haplotypes are from rs1048661-rs3825942-rs2304722.

Previous data on adipose tissues have shown that the expression of *LOXL1* is decreased by 7.7% per risk allele of SNP rs1048661 (R141L) in PEXS and pseudoexfoliation glaucoma [8]. To verify if the associated haplotypes could affect the expression levels of *LOXL1*, independently from the rs1048661 genotype, we compared the lymphocyte expression patterns of *LOXL1* between patients homozygous for risk, protective and neutral haplotype. We performed a quantitative real-time reverse transcription PCR (qRT-PCR) assay on lymphocyte cells from nine healthy samples. Three samples were homozygous for the neutral haplotype, three samples were homozygous for the protective haplotype and three samples were homozygous for the risk haplotype.

**Figure 2:**
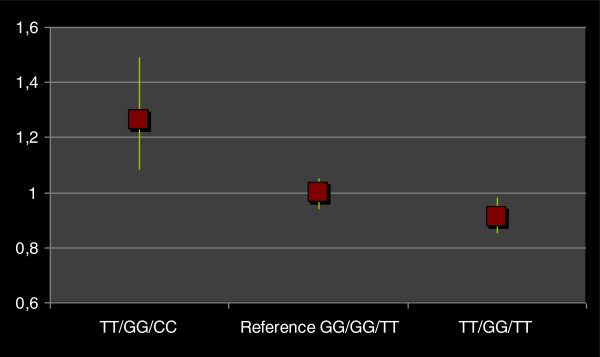
***LOXL1 *mRNA expression in lymphocytes of samples homozygous for risk haplotype (TGC), neutral haplotype (GGT) and protective haplotype (TGT).** LOXL1 mRNA expression in “at risk individuals” and “protective individuals” was respectively lower 0.91 (95% C.I. 0,85-0,98) C.I. and higher 1,26 (95% C.I. 1,08-1,49) than that in individuals harbouring neutral (non associated) haplotype 1,00 (95% C.I. 0,94-1,05). Beta 2-tubulin was used to normalize for any differences in mRNA.

Expression analysis revealed that samples harbouring the risk haplotype showed a decrease of expression levels of *LOXL1* (9%) with respect to the neutral (unassociated) haplotype. Interestingly, expression levels of *LOXL1* increased in the sample homozygous for the protective haplotype (26%) with respect to the samples harbouring neutral haplotypes (Figure 2).

## Discussion

We found that common sequence variants of *LOXL1* gene are associated with an increased risk of PDS/PG. Our results are in accord with the findings of Rao and colleagues [15] in that there is no significant association for the *LOXL1* SNPs (rs1048661 and rs3825942) with PEXG in PG/PDS only [8].

In our study, the full sequencing of the *LOXL1* gene allowed us to identify that a different sequence variation, rs2304722, is strongly associated with PDS/PG.

Markers rs1048661 and rs3825942 are statistically associated with the disease only when combined with rs2304722. Allele and haplotype associations were also supported by expression analysis, revealing the existence of a differential regulation of *LOXL1* mRNA in samples harbouring the risk or protective haplotypes. These data suggest that the downregulation of *LOXL1* is independent from the rs1048661 genotype and is associated with the susceptibility to PG/PDS. It should also be outlined that the association of rs2304722 might reflect the presence of a genuine association owing to LD with another, putatively causal polymorphism in the adjacent *LOXL1* gene region. The genetic association of *LOXL1* SNPs and the expression analysis in PDS/PG support our hypothesis of a functional role of the product of the *LOXL1* gene in the pathogenesis of pigmentary disorders. The expression levels of *LOXL1* were determined on c-DNA from peripheral blood lymphocytes by quantitative real-time RT-PCR, since it is difficult to obtain tissue from eye tissues.

The *LOXL1* gene, a member of the lysyl oxidase family, consists of seven exons, five of which (exon 2–exon 6) are of similar size and encode proteins with 76% amino acid identity. 16 Exon 1 codes for a signal peptide, a putative pro-enzyme region, and the beginning of a mature enzyme [16]. Lysyl oxidases are extracellular copperrequiring enzymes that catalyse the cross-linking of collagen and elastin through the oxidative deamination of a lysine or hydrolysine side chain, with the consequential formation of elastin polymer fibres [16,17]. The elastic fibres are components of the extracellular matrix and confer resilience [16].

Liu et al. showed that mice lacking *LOXL1* do not deposit normal elastic fibres in the uterine tract postpartum and develop pelvic organ prolapse, enlarged airspaces of the lung and loose skin and vascular abnormalities with concomitant tropoelastin accumulation [18].

## Conclusion

The pathogenesis of PG has not yet been characterised, and it has been hypothesised that a major role is exerted by the presence of iris concavity, which leads to iridozonular friction and iris pigmented epithelium disruption. The findings of our study support the hypothesis of the presence of defects in the stromal iris elastic fibres linking to the mutation of the *LOXL1* gene that could justify the presence of the increased iris concavity frequently presented in PDS/PG. The results of this work, which need to be confirmed by independent studies, suggest new insights on the molecular basis of iris pigment dispersion and related glaucoma, with presumed benefits for the screening and management of diseases.

## Competing interests

The authors declare that they have no competing interests.

## Authors’ contributions

EG, FO, MC, LT, GN and GM designed the study. LT, FO, MC collected data. TL, CP carried out the laboratory procedures. FO, MC, LT performed data analysis. EG, FO drafted the manuscript. All authors revised the manuscript. All authors read and approved the final manuscript.

## Pre-publication history

The pre-publication history for this paper can be accessed here:

http://www.biomedcentral.com/1471-2415/14/52/prepub
